# COVID-19 Recombinant mRNA Vaccines and Serious Ocular Inflammatory Side Effects: Real or Coincidence?

**DOI:** 10.18502/jovr.v16i3.9443

**Published:** 2021-07-29

**Authors:** Arash Maleki, Sydney Look-Why, Ambika Manhapra, C. Stephen Foster

**Affiliations:** ^1^Massachusetts Eye Research and Surgery Institution, Waltham, MA, United States; ^2^The Ocular Immunology and Uveitis Foundation, Waltham, MA, United States; ^3^Harvard Medical School, Department of Ophthalmology, Boston, MA, United States

**Keywords:** Acute Zonal Occult Outer Retinopathy, Antibody, Arteritic Anterior Ischemic Optic Neuropathy, AZOOR, COVID-19, GCA, Giant Cell Arteritis, SARS-CoV-2, T-helper 1

## Abstract

**Purpose:**

To report two cases; bilateral arteritic anterior ischemic optic neuropathy (AAION) and bilateral acute zonal occult outer retinopathy (AZOOR) after COVID-19 mRNA vaccination.

**Case Report:**

The first patient was a 79-year-old female was presented to us 35 days after a sudden bilateral loss of vision, which occurred two days after receiving the second recombinant mRNA vaccine (Pfizer) injection. Temporal artery biopsy was compatible with AAION. At presentation, the best-corrected visual acuity was 20/1250 and 20/40 in the right and left eyes on the Snellen acuity chart, respectively. There was 3+ afferent pupillary defect in the right eye. The anterior segment and posterior segment exams were normal except for pallor of the optic nerve head in both eyes. Intraocular pressure was normal in both eyes. She was diagnosed with bilateral AAION and Subcutaneous tocilizumab 162 mg weekly was recommended with monitoring her ESR, CRP, and IL-6.

The second patient was a 33-year-old healthy female who was referred to us for a progressive nasal field defect in her left eye, and for flashes in both eyes. Her symptoms started 10 days after receiving the second recombinant mRNA vaccine (Moderna) injection. Complete bloodwork performed by a uveitis specialist demonstrated high ESR (25) and CRP (19) levels. As a result, she was diagnosed with unilateral AZOOR in her left eye and was subsequently treated with an intravitreal dexamethasone implant in the same eye. At presentation, vision was20/20 in both eyes. The anterior segment and posterior segment exams were completely normal except for the presence of abnormal white reflex in the temporal macula of her left eye. We diagnosed her with bilateral AZOOR. Since she was nursing, intravitreal dexamethasone implant was recommended for the right eye.

**Conclusion:**

There may be a correlation between ocular inflammatory diseases with autoimmune mechanism and the mRNA COVID-19 vaccination.

##  INTRODUCTION

A new coronavirus (CoV) was discovered in December 2019 in Wuhan, Hubei province, China. This virus was called SARS- CoV-2 due to the genomic similarities to SARS-CoV, that caused severe acute respiratory syndrome (SARS). SARS-CoV2 rapidly spread all around the world and was deemed the COVID-19 pandemic by the World Health Organization (WHO). Currently, this pandemic has caused confirmed infections in over 160 million people and over 3 million deaths.^[1]^

Vaccination remains one of the most effective interventions in human history against different viral infections. Nowadays, novel recombinant vaccines are preferred instead of live, weakened, or inactivated virus vaccines as they may have greater efficacy and increased response predictability.^[2]^ In December 2020, the Food and Drug Administration (FDA) approved two vaccines for prevention of COVID-19 infection. Potential side effects of the vaccines were reported; these included mild symptoms such as pain at the site of infection, fatigue, myalgia, and fever, and more serious symptoms such as septic shock.^[2]^

Although neurological side effects of COVID-19 vaccination have been discussed,^[2]^ to the best of our knowledge, serious ocular complications of the FDA approved recombinant vaccine (mRNA) have not been reported. In these presented cases, we discuss our observation of two patients who developed arteritic anterior ischemic optic neuropathy (AAION) and bilateral acute zonal occult outer retinopathy (AZOOR) after their vaccination.

##  CASE REPORTS

### Case 1

**Figure 1:**
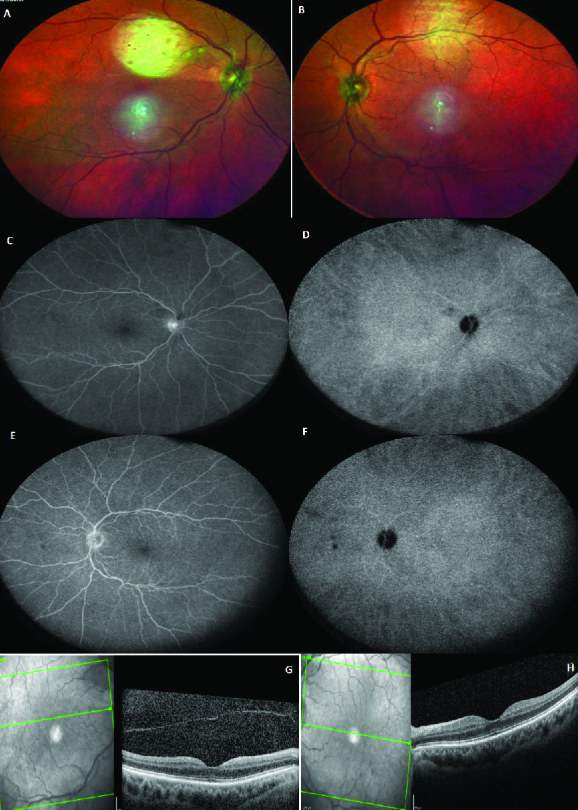
**A **and** B** are multicolored images of the right and left eyes, respectively, which illustrate generalized disc pallor in the right optic nerve head and inferior pallor in the left optic nerve head. **C** and **D** are the green reflectance images of the right and left eyes, respectively, which demonstrate generalized nerve fiber loss in the right eye and inferior nerve fiber layer loss in the left eye. **E** and **F** are the late phase of fluorescein angiography of the right and left eyes, respectively, and **G** and **H** are macular optical coherence tomography of the right and left eyes, respectively. These appear normal.

**Figure 2:**
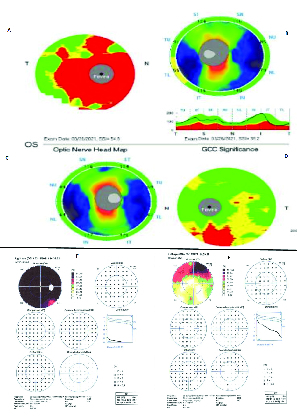
**A** and **B** illustrate ganglion cell complex (GCC) and retinal nerve fiber layer (rNFL) thickness of the right eye, respectively. GCC image demonstrates significant thinning (red area), and rNFL looks normal. **C** and **D** show rNFL thickness and GCC of the left eye, respectively. GCC image demonstrates thinning inferiorly (red area)andrNFL looks normal. **E** and **F** are Octopus EyeSuite ${}^{TM}$ Static perimetry V6.3.1 visual field of the right and left eyes, respectively. Right eye visual field shows severe generalized depression, and the left eye depicts a superior altitudinal defect.

**Figure 3:**
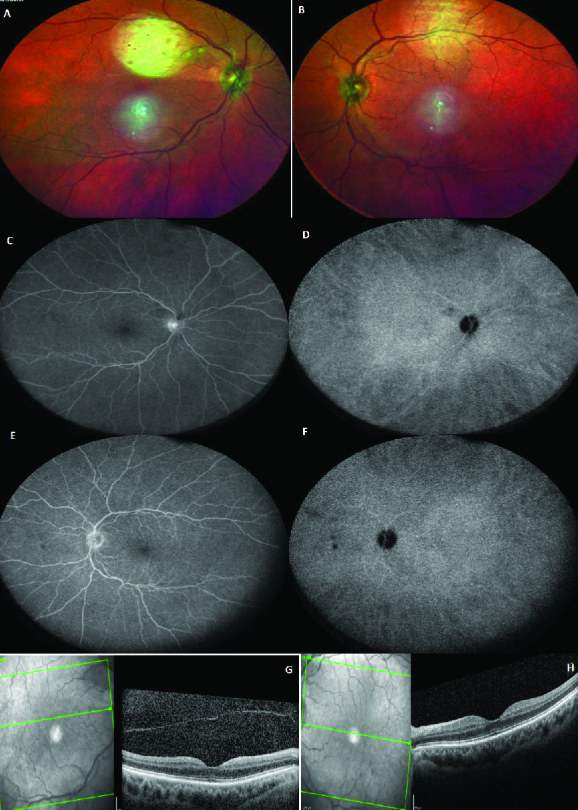
**A** and **B** illustrate fixed-luminance electroretinography (ERG) on Diopsys, which is equal to 30-Hz flicker conventional ERG. Waves have normal magnitude (amplitude) and phase (implicit time) in both eyes. Graphs show rays (blue lines) in the green area, which indicates a normal pattern. **C** and **D** show multifocal ERG of the right and left eyes, respectively. Multifocal ERG in both eyes is normal.

Thefirst patient was a 79-year-old female, otherwise healthy, with a history of osteoporosis and osteoarthritis. She was presented to us 35 days after a sudden bilateral loss of vision, which occurred two days after she received the second recombinant mRNA vaccine (Pfizer) injection. The patient did not have any other accompanying systemic or ocular symptoms. She also had no family history of autoimmune diseases. Prior to presentation at our clinic, the patient was hospitalized for very high erythrocyte sedimentation rate (ESR) and C-reactive protein (CRP) levels (i.e., ESR: 61 and CRP: 32). The patient was on 50 mg of oral prednisone for only one day. As a result of the high ESR and CRP, she underwent a right temporal artery biopsy that was compatible with AAION and was simultaneously started on high dose intravenous steroid pulse therapy (1 g) for three consecutive days. She was then administered 60 mg of oral prednisone with a slow taper. When we eventually saw the patient, she was on oral prednisone at 40 mg/day. At presentation, the best-corrected visual acuity was measured at 20/1250 and 20/40 in the right and left eyes on the Snellen acuity chart, respectively. There was 3+ afferent pupillary defect in the right eye. The anterior segment and posterior segment exams were normal except for mild cataracts in both eyes, complete pallor of the optic nerve head in the right eye, and segmental (inferior) pallor in her left eye [Figure 1].Intraocular pressure was normal in both eyes. Figure 1 also illustrates the macular optical coherence tomography (OCT), fluorescein angiography (FA), and indocyanine green angiography (ICG) in both eyes. These tests demonstrate generalized disc pallor in the right optic nerve head and inferior pallor in the left optic nerve head. Figure 2 illustrates the retinal nerve fiber layer (rNFL) and ganglion cell complex (GCC), and Octopus EyeSuite Static perimetry V3.6.1 in both eyes. The ganglion cell complex is thin (red area) in both eyes. The retinal nerve fiber layer thickening is within the normal limit in both eyes. The right eye visual field shows severe generalized depression and the left eye depicts superior altitudinal defect**. **Figure 3 illustrates the fixed luminance- (FL-) and multi luminance- (ML-) flicker electroretinography (ERG) and multifocal- (MF)ERG in both eyes, both of which are normal. Based on her diagnosis, we started the patient on 162 mg of weekly subcutaneous tocilizumab with plans to taper the steroid slowly while monitoring her ESR, CRP, and IL-6. The efficacy of the therapy will be evaluated in three months.

**Figure 4:**
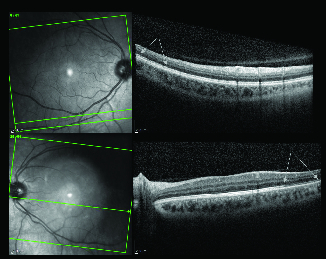
The upper and lower pictures are macular optical coherence tomography of the right and left eye, respectively. Arrows show the areas of disruption and segmentation of the ellipsoid zone in the right eye and thinning of (absent in some areas) ellipsoid zone in the left eye.

**Figure 5:**
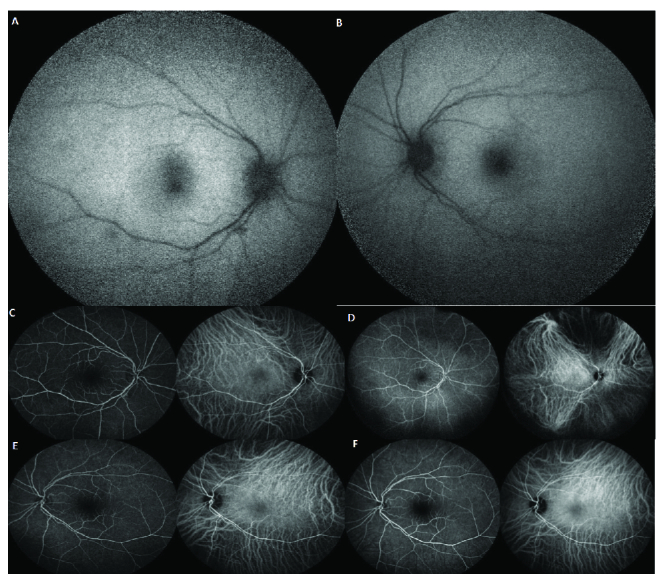
A and B illustrate fundus autofluorescence (FAF) of the right and left eyes, respectively. These images appear normal. C and D are early and late images of fluorescein angiography and indocyanine green angiography of the right eye, respectively. They are within the normal limit. E and F are early and late images of fluorescein angiography and indocyanine green angiography of the left eye, respectively. They are within the normal limit.

**Figure 6:**
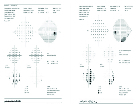
The left and right images are Humphrey visual field 24-II of the right and left eyes, respectively. The right eye visual field is reliable and shows a nonspecific nasal defect. The left eye visual field is unreliable and shows an impressive nasal field defect.

**Figure 7:**
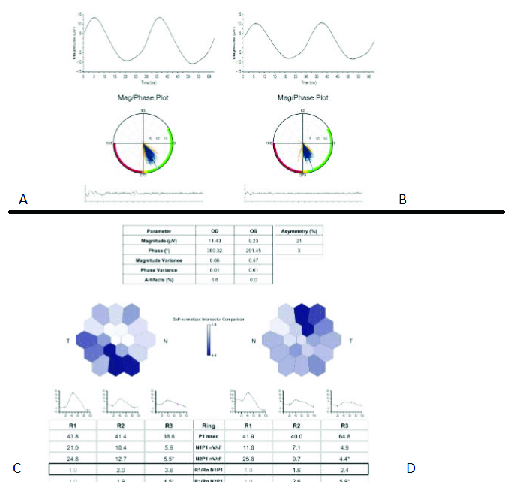
A and B illustrate fixed-luminance electroretinography (ERG) on Diopsys, which is equal to 30-Hz flicker conventional ERG. Waves have normal magnitude (amplitude) and phase (implicit time) in both eyes. Graphs show rays (blue lines) in the green area that means within the normal limit. C and D show multifocal ERG of the right and left eyes, respectively. C shows abnormal area inferotemporal correspondents with ellipsoid zone segmentation and disruption on macular optical coherence tomography. D shows abnormal areas in temporal and supratemporal correspondent with ellipsoid zone thinning (absent in some areas) on the macular optical coherence tomography.

### Case 2

A 33-year-old healthy female was referred to us for a progressive nasal field defect in her left eye and photopsia in both eyes. Her problem started 10 days after receiving the second recombinant mRNA vaccine (Moderna) injection. Prior to her last pregnancy, the patient had two episodes of preeclampsia and one unexplained miscarriage. She has no family history of autoimmune diseases. The patient initially saw one general ophthalmologist, and then a retinal specialist. She was then referred to a uveitis specialist, who discovered from the macular OCT test, outer retinal layer segmental disruption in her left eye. All of the blood work, which was completed by the uveitis specialist, demonstrated high ESR (25) and CRP (19). As a result, she was diagnosed with unilateral AZOOR in her left eye and was subsequently treated with an intravitreal dexamethasone implant in this same eye.

The patient was referred to us for a second opinion, and at presentation, had 20/20 vision in both eyes. The anterior segment and posterior segment exams were completely normal except for a yellow–white reflex in the temporal macula of her left eye. There was no inflammation in either the anterior chamber or the vitreous cavity. Intraocular pressure was normal in both eyes. Figure 4depicts the macular OCT of both eyes, which shows a segmented and disrupted ellipsoid zone in the right eye and a very thin ellipsoid zone (absent in some areas) in her left eye. Figure 5demonstrates fundus autofluorescence (FAF), FA, and ICG in both eyes, which appear to be normal. Figure 6depictsthe Humphrey visual field test results in both eyes. The right eye visual field is reliable and shows a nonspecific nasal field defect. The left eye visual field is unreliable and shows an impressive nasal field defect. Figure 7 depicts FL- and ML-flicker ERG and MF-ERG in both eyes. From the MF-ERG, defective areas were observed in the inferotemporal macula and temporal macula in the right and left eyes, respectively. Based on the test results we diagnosed her with bilateral AZOOR and recommended a combination therapy of azathioprine and cyclosporine; however, the patient preferred to first consult with her obstetrician/gynecologist prior to starting therapy as she is nursing a three-month-old baby. We believe that her intravitreal dexamethasone implant should control her inflammation while she is nursing. We also recommend that her disease activity be re-evaluated after nursing is stopped to determine what immunomodulatory therapy (IMT) is required.

##  DISCUSSION

Vaccinations have been the most effective method for prevention of infectious diseases in the past century.^[3]^ Although they are usually safe, neurological disorders (e.g., Guillain Barre syndrome [GBS], multiple sclerosis), articular disorders (e.g., arthritis, rheumatoid arthritis), and other autoimmune diseases such as systemic lupus erythematosus (SLE) have been reported as side effects after injection of varied types of vaccines.^[4]^

The etiology of the occurrence of autoimmune diseases after receiving vaccination is unclear.^[5]^ However, the possible mechanisms that may trigger these occurrences are as follows:

(i) Molecular mimicry; where the vaccine triggers an immune response to self-antigens (e.g., antibody and/or T-cells cross-reaction with self-antigens);

(ii) Bystander activation of sequestered self-antigens from the host that can activate antigen-presenting cells and T-helper cells (autoreactive T-cells);

(iii) Cytokines secretion from macrophages that recruit additional T-helper cells;^[6]^

(iv) Possible genetic background (genetic polymorphisms related to the inappropriate regulation of the IL-4 expression and/or the activity of IL-4 cytokine both of which may over-stimulate inflammatory responses).^[7]^

As a matter of comparison, it has been reported that there exists some correlation between the influenza vaccine with diabetes and the GBS; the hepatitis B vaccine with multiple sclerosis and SLE; the measles-mumps-rubella vaccine with rheumatoid arthritis and idiopathic thrombocytopenia; and the human papilloma virus with SLE, ovarian failure, and transverse myelitis.^[6]^

It is also noted that two cases of multiple evanescent white dot syndrome (MEWDS) have also been reported post human papilloma virus and rabies vaccination.^[8,9]^ Giant cell arteritis (GCA) occurring after receiving the influenza vaccination has also been documented in previous studies.^[10]^

Over the past decade, mRNA has become a promising tool in vaccine development. These vaccines were principally designed for cancer immunotherapy and the prevention of infectious diseases.^[11]^ Newly developed mRNA vaccines are characteristically very stable. These vaccines have also shown good efficacy and safety profiles in multiple clinical trials.^[12]^

Despite the recent progress made, mRNA vaccines may give rise to a cascade of immunological events, which can eventually lead to the aberrant activation of the innate and acquired immune system.^[13]^

Prior to translation, mRNA vaccines can activate several proinflammatory pathways, including type I interferon and nuclear translation of the transcription factor, nuclear factor (NF)-kB.^[14]^ Activation of these pathways is the basis of immune-mediated diseases, especially in genetically predisposed subjects such as young females.^[15,16]^

The mRNA 1273 (Moderna Inc. Cambridge, MA, USA) was the first mRNA vaccine created against COVID-19. This vaccine uses lipid nanoparticle (LNP)-encapsulated mRNA that encodes for a full-length, prefusion stabilized S protein of SARS-CoV-2.^[17]^ BNT-162 (Pfizer BioNTech, Mainz, Germany) is the other LNP mRNA vaccine. These two vaccines are similar with respect to their mild to moderate dose-dependent side effects. Both vaccines result in producing high levels of neutralizing antibodies after injection. These neutralizing antibodies recognize and target the spike proteins in the virus, killing it before the virus is disseminated and cause illness.^[2]^

Similarities exist between the SARS-CoV-2 spike glycoprotein and human proteins.^[18]^ It has been previously confirmed that some patients who have tested positive for SARS-CoV-2 immunoglobulin G (IgG) and immunoglobulin M (IgM) antibodies have developed antinuclear antibodies (ANA), anti-double-stranded deoxyribonucleic acid (DNA), anti-actin antibodies, rheumatoid factor, and anti-mitochondrial antibodies.^[4]^

Humoral and cell-mediated immunity both play a protective role in the recovery of patients from SARS-CoV-2 infection.^[19,20]^ The apparent development of antibodies should also be applicable to the COVID-19 mRNA vaccines.

An effective vaccine against COVID-19 will likely require both neutralizing antibodies and T-helper 1-driven cellular components.^[22]^ Robust B-cell activation and proliferation have been observed in subjects after receiving COVID-19 vaccination. High IgA and IgG antibodies against SARS-CoV-2 spike protein have both been detected in sera from vaccinated volunteers.^[23]^ It has also been demonstrated that the ChAdOx1 nCoV-19 vaccine induces a broad and strong T-cell response. Analysis of cytokine secretions after SARS-CoV-2 vaccination demonstrated that IFN-γ and IL-2 secretion increased compared to controls, with no change in IL-4 and IL-13 levels. This indicates a predominantly T-helper 1 response.^[22]^ These characteristics described above should be present when determining effective COVID-19 vaccines, including mRNA ones.

Unfortunately, as mentioned earlier, narcolepsy, GBS, multiple sclerosis, demyelinating neuropathies, SLE, and postural orthostatic tachycardia syndrome in susceptible subgroups are examples of vaccine-induced autoimmune issues from autoimmune cross-reactivity.^[18]^ To date, however, GBS has been the only autoimmune issue reported as a side effect of the COVID-19 vaccine.^[2]^

It should be noted that guidelines provided by the Guillain-Barre Syndrome/Chronic Inflammatory Demyelinating Polyneuropathy Foundation (GBS-CIDP) explain that if GBS occurs within four to six weeks of receiving an immunization, it can be attributed to the vaccine; however, later than this time frame would render the vaccine an unreliable cause of GBS.^[24]^

Most studies on vaccine-related side effects reveal symptom reduction during the analysis of the 10–20 days follow-up.^[6]^ This suggests that any new finding within this time range, especially in a healthy person, can be considered as vaccine-related adverse effects until proven otherwise. With reference to the two cases presented here, both of our patients developed AZOOR and AAION within the time frames described above, so the rule is applicable.

AAION and AZOOR are both autoimmune diseases. Based on the aforementioned discussions, neutralizing antibodies against SARS-CoV-2 spike proteins and/or activated T-helper-1 cells after vaccination can cross-react with proteins and antigens in large arteries, outer retinal layers, and retinal pigment epithelial cells, causing presentation with the typical form of AAION and AZOOR. Additionally, presenting symptoms early after the second COVID-19 shot (within 10–20 days period) appears to provide further evidence that supports the direct correlation between COVID-19 vaccination and the development of these autoimmune diseases. With direct reference to our cases, the presentation of ocular diseases in both patients after receiving the second shot, which boosts the immune system, may provide additional evidence to support the correlation between vaccinations and ocular diseases. The high ESR and CRP levels further support this assertion, and can also indicate the overactivity of the immune system. It is important to note that normal FAF does not rule out AZOOR; however, its presence and pattern can be beneficial in AZOOR suspected patients.^[25,26]^

In conclusion after assessing the facts and the circumstances surrounding the two cases, we conclude that there may be a correlation between ocular inflammatory diseases with autoimmune mechanism and COVID-19 vaccination. The timing of the occurrences of the autoimmune diseases, AAION and AZOOR, in our patients coincides with the timing parameters outlined for determining possible side effects as a consequence of receiving the vaccine. In addition to which, the absence of the respective comorbidity in either of our patients prior to the vaccination also suggests a possible cause and effect scenario.

##  Financial Support and Sponsorship

Nil.

##  Conflicts of Interest

There are no conflicts of interest.
